# Emergent (branched bur-reed—*Sparganium erectum* L.) and submergent (river water-crowfoot—*Ranunculus fluitans* Wimm., 1841) aquatic plants as metal biosorbents under varying water pH conditions in laboratory conditions

**DOI:** 10.1007/s11356-023-28752-x

**Published:** 2023-07-22

**Authors:** Magdalena Senze, Monika Kowalska-Góralska, Katarzyna Czyż

**Affiliations:** 1 Department of Limnology and Fishery, Institute of Animal Breeding, Wrocław University of Environmental and Life Sciences, Ul. Chełmońskiego 38C, 51-630 Wrocław, Poland; 2 Department of Sheep and Fur Animals Breeding, Institute of Animal Breeding, Wrocław University of Environmental and Life Sciences, Ul. Kożuchowska 5A, 51-631 Wrocław, Poland

**Keywords:** Hydromacrophytes, Trace elements, Bioaccumulation, Metal pollution index

## Abstract

A laboratory experiment was performed to find out the potential bioaccumulation of metals (Al, Cu, Cd, Ni, Pb, Fe, Mn) in aquatic plants acting as natural biosorbents in the environment depending on the variation of water pH. Two plants were selected for the study: branched bur-reed—*Sparganium erectum* L., as a representative of emergent plants; and river water-crowfoot—*Ranunculus fluitans* Wimm., 1841, as a representative of submergent plants. The bioaccumulation of metals in plants relative to water (BCF_W_) was determined, and the metal pollution index (MPI) was calculated. The metal content in water and plants before the experiment was arranged in the following series of increasing values: Cd = Ni < Cu < Pb < Mn < Al < Fe (water) and Cd < Cu < Ni < Pb < Al < Fe < Mn (in both plants). The lowest concentration of cadmium was found in water both at the end and beginning of the experiment. In turn, aluminum level increased, and its concentration was the highest in both acidic and alkaline water. This suggests that aluminum is definitely more available to the organisms living in it under such conditions, and in higher concentrations may be a limiting factor for them. Bioaccumulation of metals (BCF_W_) in neutral and alkaline environments was higher in branched bur-reed, and in acidic conditions in river water-crowfoot. This may provide a basis for using specific plant species to selectively accumulate metals depending on the range of water pH. This is also supported by the MPI values, which indicate that they have a very high and often the highest impact on metal pollution levels. The observed variability of metals bioaccumulation against the condition of hydromacrophytes makes it possible to consider these plants as biosorbents used in constructed biological treatment plants, which, depending on the species placed there, can selectively absorb particular trace elements. The plants used in the experiment are common hydromacrophytes of Europe, so they can be used in the construction of artificial wetlands across the continent. The apparent diversity of metal accumulation in relation to hydromacrophytes indicates that this type of site can be inhabited by specific species, which can significantly improve the purification of natural or anthropogenic water reservoirs, i.e., those that are intended to perform a filtering function.

**Supplementary Information:**

The online version contains supplementary material available at 10.1007/s11356-023-28752-x.

## Introduction

Submergent and emergent plants are a natural part of the aquatic environment. Their number and species composition depend on the type of reservoir and chemistry of water and bottom sediments as well as on the transformations in the reservoir and catchment. However, the most important is water quality which is further reflected in the condition of living organisms inhabiting the reservoirs (Gensemer and Playle 1999; Bonanno 2011, 2012, 2013; Sáez et al. 2012). The elements necessary for the proper functioning of macrophytes include macronutrients (above 0.01%—N, P, S, Ca, Mg) and micronutrients (in the range of 0.00001–0.01%—Fe, Mn, Zn, Cu, B, Mo, Cl, Ni) (Siwek 2008). Elements with toxic properties belong to the group of heavy metals (specific gravity above 5 g⋅cm^−3^), but other elements, such as aluminum (a light metal) and arsenic (a semi-metal), also have this type of harmful character. On this basis, this group of elements is referred to as trace metals (Krzesłowska 2004). The influence of metals of geochemical, i.e., natural, origin is much weaker than artificial ones and depends on the type of metal and its concentration and form (Świderska-Bróż 1993; Adams et al. 2018). Some of the metals can have a stimulating effect on the normal course of metabolic processes (Fe, Mn, Cu, Zn, Mo), and they are then more harmful to plants than to other living organisms (Kowalska-Góralska et al. 2015; Garncarek et al. 2019; Senze and Kowalska-Góralska 2020). The second group is made up of elements (As, Hg, Pb, Cd), which even at low concentrations are toxic, but far less so to plants than to other organisms (Ociepa-Kubicka and Ociepa 2012). The series of their toxicity is as follows: Ag^+^ > Hg^2+^ > Cu^2+^ > Cr^3+^ > Pb^2+^ > Ni^2+^ > Cr^6+^ > Zn^2+^ (Świderska-Bróż 1993).

The levels of metals recorded in the hydromacrophytes of fresh flowing and standing waters are quite variable (mg∙kg^−1^): Cu—5.00–30.00; Ni—0.10–5.00; Pb—0.10–5.00; Cd—0.05–0.20; Zn—10.00–70.00; Mn—70.00–500.00; Fe—50.00–200.00; Al—10–900.00 (Market 1992; Kabata-Pendias 2010). The level of metals is different for submergent and emergent species, which can result from the movement of metals with the atmospheric air, and therefore their deposition on the above-water parts of plants (Kufel and Kufel 1984). Anthropogenic atmospheric precipitation tends to contribute the highest amounts of lead, but this is dependent on current dust. Runoff from crop fields contains mainly copper, mercury, and arsenic, while municipal and industrial wastewater contains lead, copper, cadmium, zinc, chromium, mercury, and nickel (Ozimek 1988; Siwek 2008; Kabata-Pendias 2010). The uptake of metals by plants also depends on the biological cycle of these organisms, species, and ecological type. Lower plants like mosses are characterized by less developed physiological barriers and uptake of metals takes place on a passive basis; hence, their accumulation against vascular plants will be much higher. Similarly, perennial plants will accumulate larger amounts of metals than annuals, even though they reside in the same reservoir. Aquatic plants can accumulate metals through the roots and leaves. Metals can be relatively easy incorporated in excess in plant structures, which results from an underdeveloped cuticle and thus the lack of a biological barrier. This passive absorption process is the reason for the non-selective uptake of metals. Therefore, there is an apparent relationship between the metal content of environmental components and its content in aquatic plant tissues. There is also a differentiation in metal uptake by hydromacrophytes. The uptake of cadmium, lead, and nickel occurs passively, while the uptake of copper and zinc is an active process, as plants need these elements (Ozimek 1988; Siwek 2008).

In the aquatic environment, the process of metal migration occurs under aerobic conditions, and then hardly soluble carbonates and hydroxides are formed, whilesulfides are formed under oxygen-poor but sulfur-rich conditions (Świderska-Bróż 1993; Gaillardet et al. 2014). The uptake of metals from water by living organisms is also dependent on salinity. The higher it is, the more difficult it is for metals to enter an organism (Świderska-Bróż 1993). Water pH, temperature, oxidation–reduction potential, organic matter levels, and total hardness are also important. However the most important is pH, which highly affects the migration of metals in the aquatic environment (Wood 1985; Świderska-Bróż 1993; Riba et al. 2004; Huang et al. 2017).

In the temperate climate zone, surface waters are most often characterized by a pH ranging from weakly acidic to slightly alkaline. The increase in water pH toward alkaline values (up to pH 9.00) promotes the formation of hardly soluble carbonates and metal hydroxides, which affects the reduction of metals toxicity (e.g., Zn, Cd, Cu, Hg, Pb) in water. The formation of toxic metal complexes under natural conditions, however, does not pose a particular threat to water in Poland, as such waters are rarely found in the area. The threat is more often expected from the discharge of anthropogenic pollution, which can be related to the formation of a toxic form of metals (Wood 1985; Sprenger et al. 1987; Świderska-Bróż 1993).

Analysis of the concentration of metals in water cannot be the only determinant for assessing the quality of the aquatic environment. In addition to water, abiotic components—bottom sediments and biotic components—plants and animals—are important and treated as biosorbents. Plants have been found to be more sensitive to the toxic effects of metals than animals, since the loss of chlorophyll becomes the cause of slowing down the process of photosynthesis and, consequently, their death (Świderska-Bróż 1993; Gensemer and Playle 1999; Bonanno 2011, 2012, 2013). Toxic metals (Fe, Cu, Zn, Cd, Hg, Pb, Al., Cr, Ni) cause oxidative stress in the cell through the formation of reactive oxygen species and free radicals. Lipid peroxidation is the cause of damage to cell membranes, nucleic acids, and consequently genetic changes (Krzesłowska 2004). The toxicity of metals in plants involves direct action on cells, which is evident in their replacement of specific metals: magnesium in chlorophyll and calcium in calmodulin. This results in disruption of biomolecules and changes in photosynthetic pigments. Chlorophyll is hydrolyzed, which entails closure of the stomata; the number of chloroplasts decreases, ATP production slows down and cell wall distortion occurs. As a result, the most important processes in the functioning of a plant organism such as photosynthesis, transpiration, and respiration are modified. Such processes were observed by Manios et al. (2003) for *Typha latifolia*. It is also worth noting that the ability to accumulate metals in the tissues of aquatic plants proceeds only up to a certain level. Also, environmental conditions are not always fully conducive for plants to take up metals while at the same time not dying themselves subjected to too high a dose (Ozimek 1988).

Nutrient uptake by aquatic plants depends also on their age and the season. Definitely higher amounts of metals are accumulated in plant tissues during summer, although some variation is observed for individual metals. Thus, the peak for manganese and copper is in September/October, for molybdenum in July, and for lead, cadmium, and cobalt, no such seasonal variation was found (Kufel and Kufel 1984). An environment rich in metals not always is very harmful to plants, as there is a phenomenon known as competitive absorption of metals, which means that a well-absorbed and inert metal will be absorbed first and in greater quantities than a more toxic one. Thus, the sorption of more harmful metals can be somehow eliminated or at least reduced (Świderska-Bróż 1993; Gensemer and Playle 1999; Bonanno 2011, 2012, 2013). The phenomenon of metal biosorption can be used by humans in the process of wastewater treatment (Obek and Sasmaz 2011; Gallon et al. 2004; Peng et al. 2008; Vymazal et al. 2009). However, it has been proven that filtration of pollutants is related to the plant species and often occurs differently in different parts of the plant. Cadmium, iron, copper, and cobalt are accumulated in the roots and shoots to which they can be transported. Lead accumulates almost exclusively in roots and very occasionally in shoots. Zinc, nickel, and manganese are recorded in equal amounts in roots and shoots (Siwek 2008).

Submergent and emergent aquatic vegetation is used in man-made wetlands, which are created to mimic natural wetlands. They are designed to treat water, municipal and industrial wastewater in urbanized catchments (Guittonny-Philippe et al. 2014; Saeed et al. 2021; Vymazal and Březinová, 2015; Ang et al. 2023; Shelef et al. 2013; Arliyani et al. 2021; Omondi and Navalia 2020). To a large extent, when talking about the neutralization of wastewater, one talks about the heavy load of biogenic compounds in the waters, which come from households, but also from livestock farming and production including food production. A separate issue deals with the presence of metals, which, discharged from production plants or municipal wastewater treatment plants, enrich surface waters with additional material (Ang et al. 2023; Guittonny-Philippe et al. 2014; Vymazal and Březinová, 2015). The removal of vegetation, which acts as a filter, is particularly important in carrying out such treatments. If such treatment is not carried out then metals return again to the environment as a result of plant decomposition (Rodriguez and Brisson 2016). Such targeted removal of specific metals from wastewater as a result of macrophyte activity has already been studied (Işık et al. 2020; Türker et al. 2017, 2019).

There are four species of bur-reed in Poland, of which *S. erectum* is the most common. It is a species often found in the lowlands and foothills. It inhabits standing and slow-moving waters, preferring eutrophic waters. It occurs to a depth of about 1 m, sometimes singly, but far more often in clusters. In general, worldwide, the largest concentrations of *S. erectum* are found in the northern hemisphere in the temperate climate area. Thus, individuals of this species are most often found in Europe, Central Asia, and northern Africa (POWO 2023; Szoszkiewicz et al. 2008).

*R. fluitans* is found throughout Europe, less frequently in the Iberian Peninsula in the Balkans, and in Poland, it is found in lowlands and foothills, along with the other four species of crowfoot. It prefers mesotrophic flowing waters with a stable mirror level and clear flow. It finds the most favorable conditions in larger rivers with dynamic currents (POWO 2023; Szoszkiewicz et al. 2008).

The aim of this study was to explore the potential bioaccumulation of metals (Al, Cu, Cd, Ni, Pb, Fe, Mn) in submergent and emergent aquatic plants acting as biosorbents in the environment depending on the variation of water pH under laboratory conditions.

*S. erectum* and R. *fluitans* were chosen for the study due to the fact that they are macrophytes quite commonly found in the catchments of lowland and submontane rivers in the territory of Poland, which allows assuming that, as common organisms, they will be able to perform the function of a bioindicator not only in the territory of Poland, but also in the territory of Europe and its adjacent areas. The present laboratory experiment is closely related to the field research conducted by the authors in the southwestern part of Poland, among others in the catchment area of the Bystrzyca River. It complements research involving the bioaccumulation of metals in aquatic vegetation of the area.

## Material and methods

### Material

The plants used in the study were collected from the Bystrzyca River flowing in southwestern Poland, in Lower Silesia Province. The Bystrzyca is a left-bank tributary of the Oder River. Its length is 111 km, and its basin area is 1786 km^2^. The sources of the river are located near Leszczyniec in the Stone Mountains above the village of Bartnica. The river flows into the Oder River in Wroclaw. The upper section of the river is typically mountainous, and the river valley is narrow. A dam reservoir was built on the river in Lubachow (water supply, power, and recreation function). The size of the catchment area of the Bystrzyca River above the Lubachów dam reservoir, together with its tributaries, is 130.69 km^2^ (GIOŚ, 2018).

The research material included aquatic plants: branched bur-reed—*Sparganium erectum* L.—as an emergent plant; and river water-crowfoot—*Ranunculus fluitans* Wimm., 1841 Lam., 1779—a submergent plant. Both plant species are not on the list of strictly protected species (Regulation of the Minister of Environment 2014).

### Methods

Plants were collected whole (root, stem, leaves, inflorescence) from the Bystrzyca River in August 2017, at a section from Jugowice (50° 44′ 24.765″; N 16° 24′ 30.242″ E) to the estuary of the Bystrzyca River to the Lubachow dam reservoir (50° 45′ 5.44″; N 16° 25′ 1.91″ E) (Polish Standards 2014).

Based on the research of Vorobieva et al. (2020), it was decided to use water closer to its natural composition for the experiment, rather than undertake the creation of artificial water using artificial media. At the same time, since a considerable amount of water was needed for the experiment, and it was not possible to transport such a large amount of water from the field, it was decided to use aquarium water for the study, the composition of which much more closely reflects natural environmental conditions (Table 1). This water was well oxygenated, characterized by a neutral reaction, mineralization at an average level, and hardness was within the range of moderate values, as was the content of biogenic substances.

**Table 1:** Water chemistry and metal content in water and aquatic plants and metal pollution index (MPI) before the experiment

Parameters	Unit	Water	Branched bur-reed	River water-crowfoot
			mg·kg^−1^	
		min–max $\overline{x }$±SD		
Reaction	pH	7.19–7.21		
		7.20 ± 0.01		
Electrical conductivity (EC)	µS∙cm^−1^	502–504		
		503 ± 0.82		
Dissolved oxygen (DO)	mgO_2_∙dm^−3^	9.97–10.23		
		10.10 ± 0.11		
Total hardness	mgCaCO_3_∙dm^−3^	169.22–174.73		
		171.79 ± 2.26		
Ca	mgCa∙dm^−3^	54.73–55.33		
		55.03 ± 0.30		
Mg	mgMg∙dm^−3^	10.32–11.43		
		10.91 ± 0.46		
Cl	mgCl∙dm^−3^	28.53–30.12		
		29.23 ± 0.66		
Sulfate	mgSO_4_∙dm^−3^	48.19–48.42		
		48.28 ± 0.10		
Nitrate	mgNO_3_∙dm^−3^	1.36–1.76		
		1.52 ± 0.17		
Al	mg∙dm^−3^	0.0011–0.0013	600.81–600.85	362.33–363.45
		0.0012 ± 0.01	600.83 ± 0.02	362.77 ± 0.48
Cu		0.0001–0.0003	23.12–23.19	20.32–20.38
		0.0002 ± 0.01	12.15 ± 0.03	20.35 ± 0.02
Cd		0.0001–0.0002	8.69–8.79	4.99–5.08
		0.0001 ± 0.01	8.75 ± 0.04	5.03 ± 0.04
Ni		0.0001–0.0002	36.54–36.58	20.73–20.77
		0.0001 ± 0.01	36.56 ± 0.02	20.75 ± 0.02
Pb		0.0005–0.0006	80.17–81.34	36.54–37.55
		0.0006 ± 0.01	80.93 ± 0.54	37.05 ± 0.41
Fe		0.0132–0.0133	1707.14–1708.22	1405.12–1406.88
		0.0133 ± 4.71	1707.77 ± 0.46	1405.75 ± 0.80
Mn		0.0007–0.0008	4327.44–4328.85	3514.59–3515.28
		0.0007 ± 0.01	4328.33 ± 0.63	3515.03 ± 0.31
MPI		0.0005	145.75	105.04

Plants from the field were transported to the laboratory in plastic containers previously washed with distilled water, filled with river water, in which they were kept until the experiment began. At the collection site, plants were washed with river water and then placed in containers with river water. In the laboratory, the plants were placed in tanks for 2 weeks, which, in addition to river water, were supplemented with aquarium water in a 1:1 ratio, with a gradual increase in the amount of aquarium water.

Seven aquaria (nos. 1–7) with a capacity of 30.00dm^3^. The pH of the water used for the study was initially pH 7.20 (Table 1). Based on the correction of the pH, waters with different water pH were obtained: aquarium no. 1—pH 2.93; aquarium no. 2—pH 4.01; aquarium no. 3—pH 5.39; aquarium no. 4—pH 6.45; aquarium no. 5—pH 7.54; aquarium no. 6—pH 8.32; aquarium no. 7—pH 9.11. The pH of water prepared for the experiment was corrected with 35–38% HCl (Sigma-Aldrich) and 25% NaOH (Sigma-Aldrich). The water pH was set at the beginning of the experiment and was not adjusted until the end of the experiment. This was a deliberate procedure to reflect the spot, one-time discharge of contaminants. The values of the pH and, at the same time, the electrolytic conductivity changed over time, as presented in Table 2 (in the supplementary material). Artificial light conditions prevailed in the aquaria for 13 h a day, based on the length of the current summer sunlight day. Water temperature was maintained at 18–20 °C. The experiment was performed in triplicate. First, the metal content of the water and plants was determined before the experiment began (Table 1). A total of 54 plants were analyzed. At weekly intervals over a period of 7 weeks, the pH and electrolytic conductivity of the water and the concentration of metals were measured in the aquaria.

During the study, 147 water samples were taken. The results were given in mg·dm^−3^.

After 7 weeks, the plants were removed from the aquaria, dried at room temperature to an air-dry state, and then cut, crushed, and homogenized. To determine the content of metals (Al, Cu, Ni, Cd, Pb, Fe, Mn), 0.5 g of air-dry sample was weighed in a Teflon dish HP-500. Then, 10 cm^3^ of concentrated HNO_3_ (Sigma-Aldrich) was added and left at room temperature for 24 h. After this time, the samples were placed in a microwave oven (Mars 5, CEM, USA) conducting a 3-stage mineralization (Table 2). The recovery of elements ranged from 92% (for Al) to 102% (for Ni). Repeatability, expressed here as relative standard deviation (RSD) of reference material, ranging from 0.021 to 0.058. The concentrations of the studied elements indicated in the blank tests were taken into account in the final results for the respective elements. After cooling to room temperature, the mineralizates were transferred to test tubes and diluted with distilled water to 25 cm^3^. Only 36 plant samples were taken, from aquariums 2–7, as the plants in aquarium 1 decayed before the end of the experiment. The results are given in mg∙kg^−1^ dry weight.

**Table 2:** Scheme of 3-stage mineralization in Mars 5 microwave oven

Stage	Power [w]	% of power	Rising time [min]	Pressure PSI	Temperature [°C]	Holding time [min]
1	300	100	10	70	110	5
2	600	100	10	100	140	10
3	600	100	15	180	180	15

The following physical and chemical properties of water were determined:

- pH by potentiometric method using pH meter CPC-411 ELMETRON (Polish Standards 2012);
- Electrolytic conductivity by conductometric method using ELMETRON CPC-411 conductivity meter (Polish Standards 1999);
- Total aluminum by electrothermal atomic absorption spectrometry (ETAAS) (Polish Standards 2002);
- Nickel, copper, zinc, cadmium, and lead by atomic absorption spectrometry with flame atomization (Polish Standard 2014);
- Iron and manganese by spectrophotometric method (Polish Standards 2001, 2016).

Metal accumulation in aquatic plants was determined by the metal bioaccumulation factor BCF_W_ as the ratio of the metal content in the aquatic plant C_P_ to its concentration in water C_W_ (Jezierska and Witeska 2001).

$${\mathrm{BCF}}_{\mathrm{W}}=\frac{{C}_{P}}{{C}_{W}}$$

The assessment of the state of plant contamination with metals was carried out using the metal pollution index (MPI) (Usero et al. 1997).

$$\mathrm{MPI}={\left({\mathrm{Cf}}_{1}\times {\mathrm{Cf}}_{2}\dots {\mathrm{Cf}}_{\mathrm{n}}\right)}^{1/\mathrm{n}}$$

where Cf_1_, Cf_2_…Cf_n_—concentration of first metal, second metal, *n*th metal.

MPI values less than 2 indicate no impact on pollution degree, values 2–5 very low impact, 5–10 low impact, 10–20 medium impact, 20–50 high impact, 50–100 very high impact, and above 100 the highest impact.

The results were verified using certified reference materials:

- Water—Trace Metals QCI—049- 1—R.T. Corporation—Larame, USA;
- Aquatic plants—IAEA-336—International Atomic Energy Agency—Analytical Quality Control Services Austria and CRM 482—Commission of the European Communities, Community Bureau of Reference—BCR.

### Analysis of the results

Analysis of the results was performed using Microsoft Office Excel 2019 and Statistica 13.0. Calculations were performed using R version 3.6.0. The Shapiro–Wilk test was performed to verify the normality of the distribution.

Spearman’s correlations were used due to the distribution of samples. Spearman’s correlation was calculated in the Statistica program, and box and whisker plots were also created in this program. All statistically significant differences were calculated at *p* < 0.05. Due to the data being defined as having a non-normal distribution, the Kruskal–Wallis test with post hoc analysis was used. An attempt was made to determine the value allowing the data to be divided into two groups differing in a statistically significant manner. The results are presented when such a value could be determined.

The PCA test using *r*-statistics was applied in order to visualize the differences between the groups (RStudio Version 1.1.442—© 2009–2018, RStudio, Inc.). It was performed on the basis of all data and presented: differences in the parameters of the examined plants depending on the pH of water and time.

Pearson’s correlations at *p* = 0.05 and multiple regression analysis with backward selection of variables for the examined parameters were calculated using TIBCO Statistica 13.3.0 (TIBCO Software Inc., Palo Alto, CA, USA).

## Results and discussion

### Plant condition

Aquatic plants underwent noticeable changes during the 2-month study cycle. The variation in pH affected the condition of the two species differently. The changes were already visible after the first week of the experiment. They concerned aquarium no. 1 with the most acidic water (pH 2.93), where a whitish bloom appeared on the river water-crowfoot, which can be referred to as chlorosis (Siwek 2008). It is likely that too high acidity of the environment was the cause of the plant’s deformation. The branched bur-reed showed no visible changes. At the same time, no differences in the appearance and condition of hydromacrophytes were noted in the other aquaria with different water pH.

In the second week, the condition of the river water-crowfoot deteriorated even more in aquarium no. 1 with the most acidic water. The plants became increasingly white and limp. The first sign of weakness, a white coating, began to appear also on the branched bur-reed (Siwek 2008). In the other aquariums, no changes were observed.

In the next 2 weeks, the situation was stable in all aquaria.

In the fifth week, rapid changes were observed in the condition of the plants. In aquarium no. 1 (the most acidic water), the vegetation decayed. This was probably the result of too high acidification of the water, as the water pH there remained at pH 3.18. Similar observations were made by Maessen et al. (1992) in a study of aquatic plants (*Luronium natans* (L.) Raf. and *Ranunculus ololeucos* J.Lloyd.). Čtvrtlíková et al. (2009), on the other hand, succeeded in distinguishing plants susceptible and not susceptible to acidification (pH 3.0).

At the same time, in aquarium no. 2 with an initial pH of 4.01 and currently pH 6.48, new leaves began to appear in both plant species, and algae developed. In turn, in aquarium no. 3, pH 5.39 and currently pH 6.70, the plants were in much better condition, but algae were also more abundant. In aquarium no. 4, initial pH 6.45, currently pH 7.26, no changes in the plants were observed, but the amount of algae was so high that they overgrew almost the entire volume of the aquarium. In the next aquarium—no. 5, pH 7.54 and currently pH 7.57, both plants were in good condition, algae were present in small amounts, and the water was clear. In the next two aquaria—nos. 6 and 7, where the water pH was alkaline (pH 8.32 and pH 8.36), the plants were in good condition, but more algae appeared, and the water became more cloudy.

After the sixth week, the experiment was ended. The plants in aquariums 2–7 were subjected to analysis. Those from aquarium no. 1 with the most acidic water (pH 2.93) decayed earlier, and the limiting factor here was probably the very acidic water pH. It can be seen from the above that neutral conditions (pH 7.54) were the most favorable for the growth of plants of both species throughout the entire cycle of the experiment. They were most similar to the natural conditions in which these hydromacrophytes live in nature (Senze et al. 2022a, 2022b).

### Characteristics of metal content in aquatic plants prior to the experiment

Table 1 shows the content of each metal in the water and aquatic plants used for the experiment. These are the values that apply to the water before it was artificially acidified and alkalized. The series of increasing values was then as follows: for water Cd = Ni < Cu < Pb < Mn < Al < Fe, and for both aquatic plant species Cd < Cu < Ni < Pb < Al < Fe < Mn. The plants were derived from the Bystrzyca River, which has been the subject of previous studies by the authors (Senze et al. 2022a, 2022b). In their studies, metal levels were determined in reed canary grass, an emergent plant, for which the series of ascending values was as follows: Cd < Ni < Cu < Pb < Zn < Mn < Al < Fe, and for water it was Cd = Ni < Cu < Pb < Al < Mn < Fe. This allows concluding that the trend of metal accumulation regardless of species remains at a similar level.

The value of the MPI metal pollution index for the water samples equaled MPI = 0.0005 and was low enough to believe that the current level of metals does not affect water pollution (Table 1) (Usero et al. 1997). For aquatic plants, the MPI reached higher values. For branched bur-reed, it was MPI = 145.75, and for river water-crowfoot, it was lower MPI = 105.04, indicating the highest impact of metal pollution.

### Characteristics of metal concentrations in water during the experiment

The results concerning the end of the experiment are included in Table 3, and Tables 4–6 (in the supplementary material).

**Table 3:** Metal series—increasing values—on the last day of the experiment

Aquarium no	Water pH	Water	Branched bur-reed	River water-crowfoot
1	2.93	Cd < Cu < Pb < Fe < Ni < Mn < Al	No plants	No plants
2	4.01	Cd < Cu < Mn < Pb < Ni < Fe < Al	Cd < Ni < Cu < Pb < Mn < Al < Fe	Cd < Cu < Ni < Pb < Mn < Fe < Al
3	5.39	Cd < Cu < Ni < Pb < Fe < Mn < Al	Cd < Ni < Cu < Pb < Mn < Al < Fe	Cd < Ni < Pb < Cu < Mn < Al < Fe
4	6.45	Cd < Cu < Ni < Pb < Fe < Al. < Mn	Cd < Ni < Pb < Cu < Al < Fe < Mn	Cd < Ni < Cu < Pb < Al < Fe < Mn
5	7.54	Cd < Mn < Cu < Ni < Fe < Pb < Al	Cd < Ni < Pb < Cu < Al < Fe < Mn	Cd < Ni < Cu < Pb < Al < Fe < Mn
6	8.32	Cd < Mn < Pb < Cu < Ni < Fe < Al	Cd < Ni < Pb = Cu < Al < Mn < Fe	Cd < Ni < Cu < Pb < Al < Mn < Fe
7	9.11	Cd < Mn < Cu < Ni = Pb < Fe < Al	Cd < Ni < Pb < Cu < Mn < Al < Fe	Cd < Ni < Pb < Cu < Al < Mn < Fe

The uptake of metals by hydromacrophytes can occur through roots from the bottom sediment and through leaves. While in rooted plants (branched bur-reed in the present work), metals are taken up in greater amounts through the root system, in submergent plants (river water-crowfoot in the present work), this process can additionally take place through the leaves. The variation is also evident in the case of individual metals and is related to environmental factors, such as the water pH discussed in this paper, among others. Predominantly, higher concentrations of metals are recorded in the roots of plants than in other organs, but this is mainly true for emergent plants. For submergent ones, there is no such regularity. This applies mainly to cadmium, lead, and copper (Ozimek 1988).

In the water characterized by the strongest acidification—aquarium no. 1 (pH 2.93)—there was a general upward trend in the pH to the value of 3.24 lasting until week 3. Electrolytic conductivity also reached steadily higher values up to a level of 6171 µS·cm^−1^. This may have been due to the enrichment of the water with minerals from decaying plants, a process that took place quite rapidly under these conditions. In the present study, chloride, sodium, potassium, and plant pigments such as chlorophyll and pheophytin were not included in the analysis, which could increase the accuracy of the interpretation of the above results. The concentration of aluminum, copper, manganese, and iron in the water with the time increased steadily until the end of the experiment and reached a maximum level of 0.8103 mgAl·dm^−3^, 0.0504 mgCu·dm^−3^, 0.4428 mgMn·dm^−3^, and 0.1475 mgFe·dm^−3^. The concentration of nickel, cadmium, and lead, after an initial increase over a further period of time, remained at similar levels. In this case, it is most likely that there was a very rapid release of metals from the plants into the water, which, given the acidic pH of the water, became the cause of their death (Siwek 2008). The metal content of the water throughout the study cycle at the end of the experiment was lowest for cadmium and highest for aluminum (Table 3). The metal pollution index (MPI) for water was MPI = 0.0720 (Table 6). Although it increased from the initial value and was still low enough to be unaffected by metal pollution (Usero et al. 1997).

In the acidic water (aquarium no. 2), similarly to that in aquarium no. 1, the water pH showed an increasing trend up to pH 6.76 with the passage of time. Electrolytic conductivity remained at a similar level to the starting one, which was, however, lower than in aquarium no. 1. Changes in the concentration of individual metals had a similar trend as in the most acidified water. The maxima for individual metals were, respectively, in a series of ascending values: 0.0121 mgCd·dm^−3^ > 0.0202 mgCu·dm^−3^ > 0.4237 mgMn·dm^−3^ > 0.0513 mgPb·dm^−3^ > 0.0387 mgNi·dm^−3^ > 0.0804 mgFe·dm^−3^ > 0.2398 mgAl·dm^−3^. The metal pollution index reached a lower value (MPI = 0.0404) than in aquarium no. 1. It was still a low value, not affecting the level of pollution (Table 6) (Usero et al. 1997).

In aquarium no. 3 (pH 5.39), a similar trend was observed for the pH, electrolytic conductivity, and metal content as for conditions in aquaria with lower water pH. However, the MPI was lower here (MPI = 0.0324), so here, too, the level of metals had no effect on water pollution (Table 6) (Usero et al. 1997).

In the weakly acidic environment in aquarium 4 (pH 6.45), a similar trend was observed for changes in water pH as in aquaria 2 and 3. Only the content of manganese compared to other metals was highly variable. Initially, the conditions were stable; in the fourth week, strong fluctuations started. In general, the series of increasing values for metals in the water was as follows: Cd < Cu < Ni < Pb < Fe < Al < Mn (Table 3). According to Usero et al. (1997), metal pollution index (MPI) that was MPI = 0.0525 was so low that it did not pose a threat to the aquatic environment (Table 6).

In aquarium no. 5 (pH 7.54), the water pH steadily decreased until the fifth week of the experiment, up to pH 7.08, only to reach higher values again from then on up to the initial level. The direction of change in electrolytic conductivity was similar to that in aquaria 2 and 3, i.e., in highly acidified water. Here, however, the values were much lower, oscillating between 584 and 802 µS·cm^−1^. The concentration of all metals had similar variability as in aquarium no. 4 (pH 6.45) and was arranged in the following series of increasing values: Cd < Mn < Cu < Ni < Fe < Pb < Al (Table 3). The MPI in this aquarium was 0.0305, which was low enough not to have a significant impact on the metal load of the tested water (Table 6) (Usero et al. 1997).

The water pH in aquarium 6 (pH 8.32) followed a similar trend to the previously discussed conditions in aquarium 5, except that the last measurement was lower (pH 7.92) than the initial measurement (pH 8.32). The electrolytic conductivity of the water had an overall increasing trend, reaching its highest value in the seventh week (864 µS·cm^−1^). The concentrations of all metals tested showed a similar direction of variation as in aquaria 4 and 5. The series of metal contents in the water was as follows: Cd < Mn < Pb < Cu < Ni < Fe < Al (Table 6). The MPI here was 0.0292, which was even lower than in the neighboring aquarium, and therefore had no effect on the metal contamination of the examined water (Usero et al. 1997).

In aquarium no. 7 (pH 9.11), the water pH reached a much lower level—closer to neutral—from the first week of the experiment and remained so until the end of the experiment. Electrolytic conductivity was characterized by relatively stable values with a slight upward trend. The range was from 775 to 973 µS·cm^−1^. The concentration of metals in the water was characterized by similar variability as in aquarium 6. Only in the case of manganese compounds irregularity was found, but it also applied to all previously discussed environments. The series of metals in the water was arranged as follows: Cd < Mn < Cu < Ni = Pb < Fe < Al; and the MPI index according to Usero et al. (1997) was even lower, equal to 0.0259, indicating the lack of influence of metals on the level of pollution (Tables 3 and 6).

### Metals in aquatic plants and their bioaccumulation

Some of the metals present in the water environment behave synergistically with each other (increase the uptake effect of other metals), while others act antagonistically (inhibit the uptake of one metal by another or cause it to precipitate). Antagonists include copper; increased levels of which are responsible for the reduction of iron and manganese, although there may be enough of them in the water. Strong antagonism is observed between copper and zinc ions, which can undergo mutual substitution (Kabata-Pendias 2010).

**Fig. 1:**
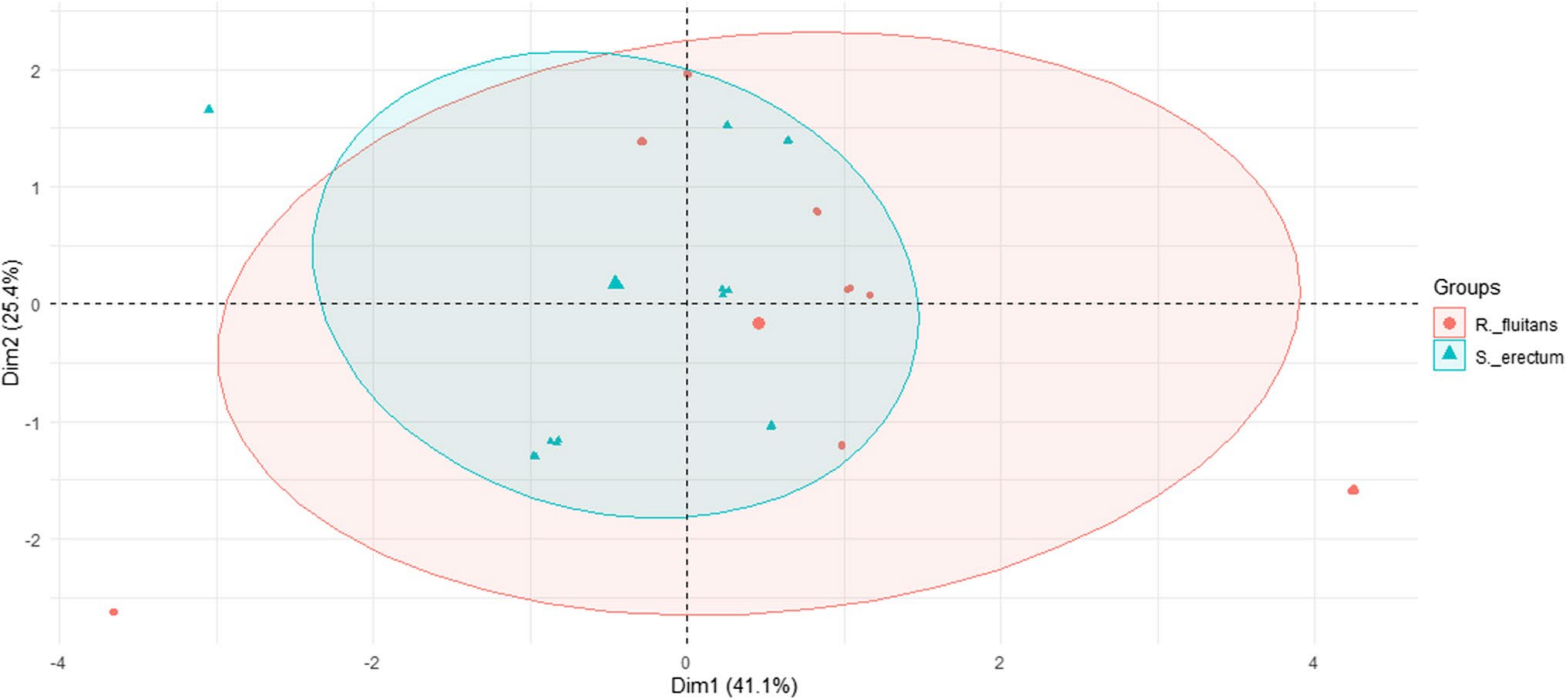
PCA plot 2D showing clustering of metal concentration in plants depending on plant species

**Fig. 2:**
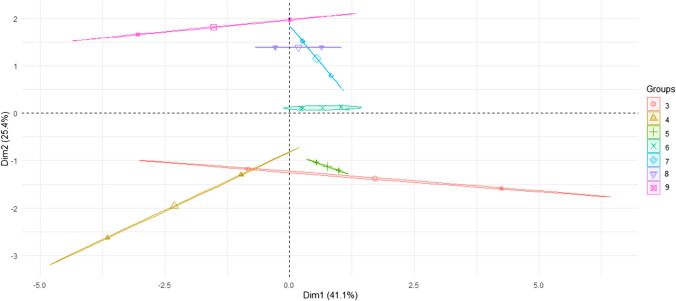
PCA plot 2D showing clustering of metal concentration in plants depending on water pH (groups)

Table 4 summarizes the results for metal levels and bioaccumulation in aquatic plants. For both plants, the trend of changes in metal content was similar in each environment, with differences mainly in metal levels. Small differences were noted between the species studied, as confirmed by the PCA analysis (Figs. 1 and 2). The species studied are representatives of submergent and emergent plants, for which one would expect clear differences in metal content, yet no such conclusion emerges from the present study. And while rooted plants accumulate larger amounts of metals (cadmium, lead, copper, and mercury) in their roots than in aboveground parts, there is no such regularity for submergent plants (Ozimek 1988). However, this thesis cannot be fully confirmed in the present study, since the plant was taken as a whole for analysis, with no distinction between root, stem, and leaves.

The metal pollution index (MPI) was determined only for plants kept in aquariums 2–7, as in the first aquarium with the most acidic water, the plants completely decayed. In aquarium no. 2 (pH 4.01), a higher MPI value was found in river water-crowfoot (MPI = 123.41)—the highest effect of pollution; and a lower value was found in branched bur-reed (MPI = 99.62)—a strong effect on pollution levels (Usero et al. 1997). In aquarium no. 3 with slightly acidic water (pH 5.39), the MPI level was equal in both plants MPI = 98 (very high impact). In the other aquaria, higher values were recorded in branched bur-reed and lower values in river water-crowfoot. In conclusion, it can be concluded that river water-crowfoot can potentially be a good plant for metal accumulation in acidic environments, and branched bur-reed in alkaline conditions. Overall, the MPI values obtained indicate that both plants have a very high and highest impact on the level of metal contamination (Usero et al. 1997).

For branched bur-reed, the highest levels of aluminum (750.88 mgAl∙kg^−1^) were recorded in aquarium no. 7, which was filled with the most alkaline water (pH 9.11), and for river water-crowfoot in aquarium no. 2 (1865.41 mgAl∙kg^−1^), where acidic conditions prevailed (pH 4.01) (Figs. 3 and 4—in the supplementary material). The overall picture for both plants in aquaria nos. 3, 4, 5, and 6 and in no. 7 for river water-crowfoot was stable, indicating that only large amounts of aluminum were present in river water-crowfoot in acidic water. Perhaps they originated from water in which aluminum concentrations were significantly higher. In the comparison of aluminum content in plants at the beginning of the experiment and at the end, it is clear that at acidic and weakly acidic conditions, the amount of aluminum in the plant decreased in branched bur-reed, while in the case of river water-crowfoot, it increased. This is reflected in the value of the bioaccumulation index (BCF_W_ = 8754.43). Under neutral and weakly alkaline conditions, aluminum levels were lower in both species at the end of the experiment. In an alkaline environment (pH 9.11), branched bur-reed accumulated higher amounts of aluminum, and river water-crowfoot accumulated lower amounts against the initial values. From the point of view of physiological transformations in plants, aluminum is among the elements that, if taken up by the plant in very small amounts, can be toxic to it (Siwek 2008). Thus, the described course of the experiment and the artificially created very acidic or strongly alkaline conditions highlight the extremely harmful effect of aluminum on the structure of plant organisms.

Manganese levels in both plants were similar (Figs. 3 and 4—in the supplementary material). The lowest values were recorded in acidic conditions and slightly higher in alkaline conditions. In water with a pH of 6.45, the manganese level was half that of the baseline. In water with a pH of 7.54, the final concentration of manganese was very close to the initial value. Manganese compounds were accumulated in greater amounts in the tissues of branched bur-reed than in river water-crowfoot, as evidenced by the values of the BCF_W_ bioaccumulation factor. Only in alkaline conditions, accumulation was about four times higher than under neutral and acidic conditions. The physiological criterion of toxicity takes into account a group of metals that, in excessively high concentrations, cause symptoms of toxicity. The observations made in the experiment are confirmed by the need of organisms in manganese, zinc, copper, nickel, and iron. This is because manganese is among the elements necessary for plants in their metabolism. It can be taken up in higher amounts, but up to a certain limit. Too high a concentration of it is likely to cause signs of toxicity in plants (Siwek 2008).

Iron levels in both plant species tested increased with increasing water pH, with slightly higher values in river water-crowfoot (Figs. 3 and 4—in the supplementary material). The maximum was found in the most alkaline water: 2254.58 mgFe∙kg^−1^ (branched bur-reed), 2547.45 mgFe∙kg^−1^ (river water-crowfoot). On the other hand, branched bur-reed placed in aquarium no. 6 and river water-crowfoot in aquarium no. 5 at the end of the experiment reached iron levels equal to the initial value. In summary, for iron and manganese, a similar trend of change is drawn. In acidic environments up to a pH of 6.45, lower amounts of metals were recorded in both plants at the end of the experiment than at the beginning. In neutral environments (pH 7.54), the opposite tendency was observed. Under alkaline conditions, branched bur-reed accumulated higher amounts than river water-crowfoot. In turn, river water-crowfoot contained lower amounts of these metals than before the experiment began.

The overall trend of changes in the copper content of both plant species was similar (Figs. 3 and 4—in the supplementary material). The highest values were found in aquarium no. 7: river water-crowfoot—66.62 mgCu∙kg^−1^; branched bur-reed—83.17 mgCu∙kg^−1^. The lowest levels were recorded in aquarium no. 6 (river water-crowfoot) and in aquarium no. 3 (branched bur-reed). In summary, in all environments, the values at the end of the experiment were higher than at the beginning. The exception was alkaline conditions (pH 8.32), where copper levels at the end decreased in both plant species.

The variability of cadmium, lead, and nickel levels in both plants was similar (Figs. 3 and 4—in the supplementary material). For river water-crowfoot, the maximum occurred in aquarium 2, and throughout the entire cycle of the experiment, the levels of these metals were higher than the initial value in all aquaria. For branched bur-reed, on the other hand, in the case of cadmium, the initial content (8.75 mgCd∙kg^−1^) was higher than the other concentrations except in aquarium no. 7 (9.27 mgCd∙kg^−1^). In aquaria nos. 1–6, cadmium levels ranged from 7.34 to 7.62 mgCd∙kg^−1^, indicating that the pH in the range of pH 4.01 to pH 8.32 had no significant effect on its content in branched bur-reed. Cadmium bioaccumulation was strongest for both plants under alkaline conditions. In the case of river water-crowfoot, it was lower at a level of BCF_W_ = 1998.50, while for branched bur-reed, it reached a higher value equal to BCF_W_ = 2527.70. In neutral (pH 7.54) and acidic (pH 4.01) environments, both species accumulated cadmium very similarly. In a comparison of initial and final values, it can be seen that branched bur-reed, with the exception of a strongly alkaline reaction (pH 9.11), contained less cadmium at the end than at the beginning. In contrast, the opposite was true for river water-crowfoot except for the pH of 6.45.

A similar trend of change as for cadmium was found for nickel. Strongly acidic, neutral, and alkaline conditions were observed to favor nickel accumulation in both plants. Only under conditions of pH 5.39 and pH 6.45 branched bur-reed reached lower levels at the end of the experiment, while river water-crowfoot reached higher levels. Nickel was accumulated in greater amounts in river water-crowfoot than in branched bur-reed. This concerned all environments except the most alkaline one (pH 9.11), for which the highest value in branched bur-reed was BCF_W_ = 2362.0, which at the same time was the maximum for all plant samples tested. However, the lowest values were recorded in acidic and neutral environments, indicating an increasing trend from acidic conditions toward alkaline ones.

Similar observations were also made for lead, except that in this case in aquaria no. 2–7, its level was lower than the initial one (80.93 mgPb∙kg^−1^). As for cadmium, a slightly higher lead content (78.44 mgPb∙kg^−1^) was observed in aquarium no. 7 with the most alkaline water pH. The level of lead at the beginning of the experiment regardless of the water pH was always higher in branched bur-reed than at the end of the experiment, while the opposite was noted for river water-crowfoot. Accumulation of lead compounds in an acidic environment was strongest in river water-crowfoot, and in an alkaline environment in branched bur-reed. Under neutral, weakly acidic, and weakly alkaline conditions, similar bioaccumulation of lead was found in both aquatic plant species. In addition, there was a progressive increase in values with increasing water pH in both species. Lead compounds were definitely strongly accumulated in branched bur-reed in each environment. The maximum for both species was recorded in aquarium no. 7 (the most alkaline environment) and amounted to BCF_W_ = 4663.59 for branched bur-reed, and BCF_W_ = 3735.73 for river water-crowfoot.

Analyzing in general the variability of metal content in plant tissues under varying water pH, it is clear that for both species, the lowest values were recorded for cadmium compounds. The highest amounts were found for iron in branched bur-reed at acidic (pH 4.01 and pH 5.39) and alkaline (pH 8.32 and pH 9.11) pH. The same was noted for river water-crowfoot with one exception; at pH 4.01, the highest amounts were recorded for aluminum. For both species at conditions close to neutral values (pH 6.45 and pH 7.54), the highest amounts were found for manganese.

The overall picture of metal bioaccumulation in hydromacrophytes shows that both plants have very good accumulation properties due to high values of the BCF_W_ accumulation factor > 1000 (Rajfur et al. 2010). The picture of accumulation in a neutral environment was the clearest. All metals tested reached higher bioaccumulation index values in branched bur-reed than in river water-crowfoot (Table 4). In acidic conditions, higher values were more common for river water-crowfoot, and in alkaline conditions for branched bur-reed. Water pH is by far the most important with regard to the accumulation of metals in plant tissues. pH values higher than pH > 6.5 decisively reduce the level of readily soluble forms of metals, and thus inhibit metal uptake and bioaccumulation in plant tissues. An acidic water pH promotes the uptake of metals—mainly cadmium, nickel, and zinc—from water even when it is not heavily laden with pollutants (Kabata-Pendias 2010).

The process and relationships of metal release from plant tissues into water have been described in the literature and cited by Ozimek (1988) for *Elodea canadensis* Michx., *Salvinia* Set., *Azolla* Lam., and *Ceratophyllum* L. Metals deposited on the surface of plants as a result of their flushing by precipitation are incorporated into the composition of water, which directly benefits the plants. However, the main pathway is the decomposition of hydrophytes in the process of their death. Most of the metals consequently end up in the bottom sediment enriching their composition. For rooted plants, they therefore become more available from the sediment than from the water and can again be absorbed by hydromacrophytes (Siwek 2008). However, some differences specific to particular metals in their penetration into water are noted. Manganese is one that is present in elevated concentrations quite quickly, which cannot be said for copper and zinc. This variability in metal concentrations can be seen within the water samples taken from 7 aquaria, where cadmium was the least present in all aquaria regardless of water reaction, while aluminum was the most present. The exception was aquarium no. 4 (pH 6.45), which had the most manganese compounds (Table 3). Here, too, this metal compared to the others was the most variable in terms of recorded concentrations. Rapid decreases and increases in values were observed in all waters. Manganese in acidic environments tended to stay in the range of higher values, and in neutral and alkaline environments in lower values. Iron tended to take on higher levels regardless of environment, while copper more often took on lower values, mainly in acidic environments.

Increased metal content in the natural environment, which originates from human activities, as well as from natural transformations, can be tolerated to some extent by hydromacrophytes, but an excess of, for example, copper or cadmium compounds, can result in disorders in the course of photosynthesis. Among others, chloroplasts are damaged and chlorophyll levels decrease. Such observations have been recorded for *Lemna minor* L.,* E canadensis*, and emergent plants (Świderska-Bróż 1993; Gensemer and Playle 1999; Bonanno 2011, 2012, 2013). Metal contents similar to those presented in this paper at water pH 5.3 and pH 6.4 and pH 7.5, but occurring under natural conditions, were recorded in their study by Samecka-Cymerman et al. (2002) for mountain rivers on the border of Poland, the Czech Republic, and Germany. In contrast, the extreme values of pH occur naturally in the area of mine influence and indicate high acidification of the aquatic environment, and thus similar conditions to the present experimental study. Under such natural conditions of reduced water reaction, the metal content ranges found were similar both in Poland and abroad (Engleman and McDiffett 1996; Winterbourn et al. 2000; Samecka-Cymerman and Kempers 2001).

Conditions closer to neutral, but also observed in nature, occurred in mountain rivers and reservoirs of southwestern Poland, where the pH range was 6.9–8.1, and in lowland rivers (Pokorny et al. 2015; Senze et al. 2009, 2021; Klink et al. 2013, 2016; Łojko et al. 2015). The general global trend indicates that regardless of the latitude of surface water bodies, bioaccumulation of metals, depending on water pH, had a similar trend (Gaur et al. 1994; Klumpp et al. 2002; Vardanyan and Ingole 2006; Rai 2009; Bonanno 2011, 2012, 2013; Gao et al. 2016).

The plants used in this experiment are commonly found in standing and flowing surface waters, and although they are representatives of two different groups of plants, they could be used as bioindicators and at the same time biosorbents of pollutants present in Europe. The water crowfoot subjected to the experiment turned out to be more sensitive to environmental acidification. Most likely, as a submergent plant, which with its entire surface is in direct contact with the pollutants present in the water, it is more quickly affected by the changes present in the environment. Branched bur-reed was not as quickly susceptible to changes, which when they did occur, however, appeared only after a 2-week delay. This may suggest that strongly acidic conditions if they persist for a short time (here up to 2 weeks) will be noticed more quickly by the water crowfoot than by the branched bur-reed. In addition, if they persist for a short time and quickly become neutralized, they may not be noticed, and the environment will return to a less acidic state. These observations were made for strongly acidic conditions. In other artificially created conditions, no such changes in the condition of the plants were seen. Besides, both species did not tolerate strongly acidic conditions, which allows us to describe both species as good bioindicators of strong water pH.

Bioaccumulation of metals in aquatic plants forms in the following order depending on their organs: stems < leaves < rhizomes < roots (Bonanno and Lo Giudice 2010). The same observations were made for plants that grew in both natural and artificial wetlands. This leads us to conclude that the experiment performed may be a representation of natural environmental conditions.

Acidic conditions promote the accumulation of metals in wetland plants, as demonstrated in a study by Alberts and Camardese (1993) taking pH 5.0 conditions as acidic and pH 6.5 as neutral. In addition, they proved that submergent plants accumulate larger amounts of metals than emergent ones. Similar observations were also made in the present experiment.

## Conclusions

Over the course of the 7-week study, variability was observed in plant conditions and measured water parameters.

The most acidic water pH was the cause of the deformation and later decomposition of the plants observed after the first week of the experiment. In the other artificially created environmental conditions, the plants survived in fairly good condition, which leads us to believe that the water pH in the range of pH 4.00–9.00 is not so harmful that macrophytes would be destroyed.

In the case of water mineralization, in all environments, electrolytic conductivity values reached higher levels on the day the experiment ended than at the beginning.

The concentration of metals in the water filling the aquaria changed during the test cycle. On the day ending the experiment, cadmium compounds were still the lowest as at the beginning. However, aluminum levels increased, with concentrations being highest in both acidic and alkaline water. Only in an environment with a pH of 6.45 were higher amounts of manganese compounds recorded. This shows that aluminum in both acidic and alkaline conditions is definitely more available to the organisms living in it, so in higher concentrations, it may be a limiting factor for them.

In hydromacrophytes, it is clear that for both species, the lowest values were recorded for cadmium compounds. The highest levels were found for iron in branched bur-reed at acidic (pH 4.01 and pH 5.39) and alkaline (pH 8.32 and pH 9.11) conditions. The same was true for river water-crowfoot, but with the exception of pH 4.01, in which case the highest amounts of aluminum were found. The highest amounts of manganese in both species occurred under conditions close to neutral values (pH 6.45 and pH 7.54).

In general, the trend of changes in the metal content of plants was similar in each aquarium; the differences were mainly in their levels. Metal pollution index (MPI) allowed us to conclude that river water-crowfoot could potentially be a good plant with metal accumulation abilities in acidic environments, and branched bur-reed in alkaline conditions. Overall, the MPI values obtained for the plants indicate that they have a very high and often highest impact on the level of metal.

Bioaccumulation of metals in neutral environments was higher in branched bur-reed than in river water-crowfoot. In contrast, in acidic conditions, higher values were more common for river water-crowfoot, and in alkaline conditions for branched bur-reed. The plants used in the experiment, as they are commonly found in Europe, are excellent materials for potential use in artificial wetlands, which provide a place for wastewater disposal. This could provide a basis for using specific plant species to selectively accumulate metals depending on the range of water pH. In addition, a valuable observation is the different sensitivity to acidification of the studied species in terms of the passage of time. In this case, water-crowfoot responded more quickly to environmental changes. The conducted studies show the possibility of metal accumulation in differentiation into submergent and emergent plants against the background of different water pH. Artificially created conditions mimicking the natural environment and the observed variability of bioaccumulation of metals against the condition of hydromacrophytes allow treating them as biosorbents used by humans. This provides a basis for designing such artificial conditions, for example, in biological treatment plants under construction, which, depending on the placed sorbent, which will be submergent or emergent plants can selectively absorb individual trace elements. The apparent diversity of metal accumulation in relation to hydromacrophytes indicates that such sites can be inhabited by specific species, which can significantly improve the purification of natural or anthropogenic water reservoirs, i.e., those which are intended to perform a filtering function.

## Supplementary Information

Below is the link to the electronic supplementary material.

Supplementary file1 (DOCX 178 KB)

## Data Availability

All data generated or analyzed during this study are included in this published article.
