# Supplementary material for: Emergent (branched bur-reed—Sparganium erectum L.) and submergent (river water-crowfoot—Ranunculus fluitans Wimm., 1841) aquatic plants as metal biosorbents under varying water pH conditions in laboratory conditions
Source: Environ Sci Pollut Res Int. 2023 Jul 22;30(40):92053–67. doi: 10.1007/s11356-023-28752-x (PMC10447270; doi:10.1007/s11356-023-28752-x)
Supplement: Supplementary file 1 — Supplementary file1 (DOCX 178 KB) [file 11356_2023_28752_MOESM1_ESM.docx]

Table 4 The pH and electrolytic conductivity (µS∙cm^­-3^) of water in individual aquaria

| Aquarium no/pH | Data pomiaru | pH | electrolytic conductivity |
| --- | --- | --- | --- |
| no 1  pH 2.93 | 26.07 | 2.93-2.94  2.93±0.00 | 2580-2581  2580.33±0.47 |
|  | 2.08 | 2.99-3.02  3.01±0.01 | 2644-2645  2644.67±0.47 |
|  | 7.08 | 3.10-3.11  3.10±0.00 | 3850-3851  3850.33±0.47 |
|  | 14.08 | 3.17-3.18  3.18±0.00 | 3780-3781  3780.67±0.47 |
|  | 21.08 | 3.09-3.10  3.09±0.00 | 3954-3955  3954.33±0.47 |
|  | 28.08 | 3.05-3.06  3.05±0.00 | 4521-4522  4521.33±0.47 |
|  | 4.09 | 3.17-3.18  3.18±0.00 | 6170-6171  6170.33±0.47 |
|  | 11.09 | 3.23-3.24  3.23±0.01 | 6170-6171  6170.33±0.47 |
| no 2  pH 4.01 | 26.07 | 4.01-4.02  4.01±0.00 | 3870-3870  3870±0.00 |
|  | 2.08 | 4.12-4.15  4.14±0.01 | 3952-3955  3953.67±1.25 |
|  | 7.08 | 5.74-5.75  5.75±0.00 | 4680-4681  4680.33±0.47 |
|  | 14.08 | 6.41-6.42  6.41±0.00 | 5240-5242  5241±0.82 |
|  | 21.08 | 5.02-5.03  5.02±0.00 | 5048-5049  5048.67±0.47 |
|  | 28.08 | 5.12-5.13  5.12±0.00 | 4532-4533  4532.33±0.47 |
|  | 4.09 | 6.47-6.48  6.48±0.00 | 4150-4151  4150.33±0.47 |
|  | 11.09 | 6.75-6.76  6.75±0.01 | 4650-4651  4650.33±0.47 |
| no 3  pH 5.39 | 26.07 | 5.38-5.39  5.39±0.00 | 875-876  875.67±0.47 |
|  | 2.08 | 5.85-5.88  5.86±0.01 | 876-877  876.33±0.47 |
|  | 7.08 | 6.44-6.45  6.44±0.00 | 876-878  877±0.82 |
|  | 14.08 | 6.68-6.69  6.68±0.00 | 853-854  853.33±0.47 |
|  | 21.08 | 5.12-5.13  5.12±0.00 | 1466-1467  1466.33±0.47 |
|  | 28.08 | 5.80-5.81  5.80±0.00 | 1573-1574  1573.67±0.47 |
|  | 4.09 | 6.70-6.71  6.70±0.00 | 1028-1029  1028.67±0.47 |
|  | 11.09 | 6.74-6.75  6.74±0.01 | 1086-1087  1086.67±0.47 |
| no 4  pH 6.45 | 26.07 | 6.44-6.45  6.45±0.00 | 1419-1420  1419.33±0.47 |
|  | 2.08 | 6.84-6.87  6.85±0.01 | 1433-1436  1433.67±1.25 |
|  | 7.08 | 7.13-7.14  7.14±0.00 | 1440-1441  1440.33±0.47 |
|  | 14.08 | 7.31-7.32  7.32±0.00 | 1408-1410  1409±0.82 |
|  | 21.08 | 7.12-7.13  7.12±0.00 | 1596-1597  1596.33±0.47 |
|  | 28.08 | 7.02-7.03  7.02±0.00 | 1658-1659  1558.67±0.47 |
|  | 4.09 | 7.26-7.27  7.27±0.00 | 1647-1648  1647.33±0.47 |
|  | 11.09 | 7.39-7.40  7.39±0.01 | 1755-1756  1755.67±0.47 |
| no 5  pH 7.54 | 26.07 | 7.54-7.55  7.54±0.00 | 585-586  585.33±0.47 |
|  | 2.08 | 7.47-7.48  7.48±0.00 | 594-598  596.33±1.70 |
|  | 7.08 | 7.24-7.25  7.24±0.00 | 596-598  597±0.82 |
|  | 14.08 | 7.12-7.15  7.14±0.01 | 584-585  584.33±0.47 |
|  | 21.08 | 7.08-7.09  7.09±0.00 | 763-764  763.33±0.47 |
|  | 28.08 | 7.15-7.16  7.15±0.00 | 801-802  801.33±0.47 |
|  | 4.09 | 7.57-7.58  7.57±0.00 | 697-698  697.33±0.47 |
|  | 11.09 | 7.61-7.62  7.61±0.01 | 718-719  718.33±0.47 |
| no 6  pH 8.32 | 26.07 | 8.32-8.33  8.32±0.00 | 722-723  722.67±0.47 |
|  | 2.08 | 8.01-8.02  8.01±0.00 | 752-755  753.33±1.25 |
|  | 7.08 | 7.70-7.71  7.71±0.00 | 771-773  772±0.82 |
|  | 14.08 | 7.92-7.93  7.92±0.00 | 741-742  744.33±0.47 |
|  | 21.08 | 7.63-7.64  7.64±0.00 | 790-791  790.67±0.47 |
|  | 28.08 | 7.45-7.46  7.45±0.00 | 850-851  850.67±0.47 |
|  | 4.09 | 7.66-7.67  7.67±0.00 | 797-799  797.67±0.94 |
|  | 11.09 | 7.92-7.93  7.92±0.01 | 863-864  863.33±0.47 |
| no 7  pH 9.11 | 26.07 | 9.10-9.11  9.11±0.00 | 801-802  801.33±0.47 |
|  | 2.08 | 8.04-8.09  8.06±0.02 | 798-799  798.33±0.47 |
|  | 7.08 | 7.66-7.67  7.66±0.00 | 797-798  797.33±0.47 |
|  | 14.08 | 7.51-7.52  7.51±0.00 | 775-776  775.33±0.47 |
|  | 21.08 | 7.50-7.51  7.51±0.00 | 801-802  801.33±0.47 |
|  | 28.08 | 7.50-7.51  7.51±0.00 | 893-894  893.33±0.47 |
|  | 4.09 | 7.38-7.39  7.39±0.00 | 966-968  967±0.82 |
|  | 11.09 | 7.43-7.44  7.43±0.01 | 972-973  972.33±0.47 |

Table 5 Metal content in water and aquatic plants, bioaccumulation factor (BCF_W_) at the end of the experiment at week 7

| Aquarium no/pH | Metal | Water | Branched bur-reed | River water-crowfoot | Branched bur-reed | River water-crowfoot |
| --- | --- | --- | --- | --- | --- | --- |
|  |  | mg·dm^-3^ | mg·kg^-1^ | | BCF_W_ | |
| no 1  pH 2.93 | Al | 0.8102-0.8103  0.8102±0.01 | No plants | | | |
|  | Cu | 0.0345-0.0347  0.0346±0.01 |  |  |  |  |
|  | Cd | 0.0127-0.0128  0.0128±0.01 |  |  |  |  |
|  | Ni | 0.0586-0.0588  0.0587±0.01 |  |  |  |  |
|  | Pb | 0.0494-0.0495  0.0495±0.01 |  |  |  |  |
|  | Fe | 0.0512-0.0514  0.0513±0.01 |  |  |  |  |
|  | Mn | 0.1523-0.1525  0.1524±0.01 |  |  |  |  |
|  | pH | 3.23-3.24  3.23±0.01 |  | | | |
|  | electrolytic conductivity (µS∙cm^­-3^) | 6170.00-6171.00  6170.33±0.47 |  | | | |
| no 2  pH 4.01 | Al | 0.2130-0.2131  0.2131±0.01 | 545.48-546.52  545.97±0.43 | 1864.52-1865.90  1865.28±0.57 | 2559.74-2564.62  2562.42±2.02 | 8749.51-  8760.09  8754.43±4.35 |
|  | Cu | 0.0177-0.0178  0.0177±0.01 | 54.77-54.80  54.78±0.01 | 46.40-46.42  46.41±0.01 | 3077.53-3096.05  3089.31±8.36 | 2607.30-2622.60  2617.12±6.96 |
|  | Cd | 0.0105-0.0106  0.0106±0.01 | 7.43-7.45  7.44±0.01 | 8.36-8.38  8.37±0.01 | 700.94-708.57  704.12±3.24 | 788.68-798.09  792.13±4.23 |
|  | Ni | 0.0344-0.0345  0.0344±0.01 | 39.43-39.45  39.44±0.01 | 47.91-47.92  47.91±0.01 | 1143.48-1146.51  1145.40±1.37 | 1388.99-1392.73  1391.48±1.77 |
|  | Pb | 0.0317-0.0318  0.0317±0.01 | 72.43-72.45  72.44±0.01 | 81.17-81.18  81.17±0.01 | 2277.99-2285.49  2282.78±3.40 | 2552.83-2560.57  2557.99±3.65 |
|  | Fe | 0.0402-0.0404  0.0403±0.01 | 885.55-885.59  885.57±0.02 | 974.52-974.56  974.54±0.02 | 21920.30-22029.60  21974.61±44.63 | 24121.78-24242.54  24182.32±49.30 |
|  | Mn | 0.0212-0.0213  0.0212±4.71 | 172.95-172.96  172.96±0.01 | 158.74-158.78  158.76±0.02 | 8120.19-8158.49  8145.57±17.95 | 7452.58-7489.62  7476.80±17.14 |
|  | pH | 6.75-6.76  6.75±0.01 |  |  |  |  |
|  | electrolytic conductivity (µS∙cm^­-3^) | 4650.00-4651.00  4650.33±0.47 |  |  |  |  |
| no 3  pH 5.39 | Al | 0.2427-0.2428  0.2427±0.01 | 440.19-440.52  440.33±0.14 | 493.54-493.77  493.66±0.09 | 1812.97-1815.08  1814.03±0.86 | 2033.54-2034.03  2033.74±0.21 |
|  | Cu | 0.0170-0.0171  0.0170±0.01 | 54.33-54.36  54.34±0.01 | 62.30-62.33  62.32±0.01 | 3177.19-3197.65  3190.44±9.38 | 3644.44-3666.47  3658.54±9.99 |
|  | Cd | 0.0053-0.0054  0.0053±0.01 | 7.41-7.42  7.42±0.01 | 5.77-5.78  5.78±0.01 | 1374.07-1400.00  1390.73±11.80 | 1068.52-1090.57  1083.22±10.39 |
|  | Ni | 0.0195-0.0196  0.0196±0.01 | 29.91-29.93  29.92±0.01 | 31.77-31.79  31.78±0.01 | 1526.02-1534.36  1529.14±3.71 | 1620.92-1630.26  1624.20±4.29 |
|  | Pb | 0.0215-0.0216  0.0215±0.01 | 60.03-60.05  60.04±0.01 | 58.52-58.53  58.52±0.01 | 2780.09-2792.56  2788.25±5.77 | 2709.72-2717.81  2717.81±5.72 |
|  | Fe | 0.0280-0.0283  0.0281±0.01 | 869.14-869.17  869.15±0.01 | 905.33-905.36  905.34±0.01 | 30712.01-31040.70  30894.68±136.67 | 31991.52-32333.21  32181.08±141.98 |
|  | Mn | 0.0344-0.0345  0.0344±0.01 | 318.26-318.28  318.27±0.01 | 302.44-302.46  302.45±0.01 | 9225.22-9252.33  9243.09±12.64 | 8766.96-8792.15  8783.66±11.81 |
|  | pH | 6.74-6.75  6.74±0.01 |  |  |  |  |
|  | electrolytic conductivity (µS∙cm^­-3^) | 1086.00-1087.00  1086.67±0.47 |  |  |  |  |
| no 4  pH 6.45 | Al | 0.2400-0.2402  0.2401±0.01 | 498.57-498.71  498.64±0.06 | 335.94-336.58  336.33±0.28 | 2076.23-2077.38  2076.80±0.47 | 1398.58-1402.79  1400.79±1.62 |
|  | Cu | 0.0164-0.0135  0.0164±0.01 | 64.15-65.55  64.98±0.60 | 54.96-55.47  55.18±0.21 | 3887.88-3996.95  3954.49±47.69 | 3330.91-3382.32  3358.07±21.09 |
|  | Cd | 0.0074-0.0075  0.0074±0.01 | 7.52-7.55  7.54±0.01 | 6.21-6.56  6.44±0.16 | 1002.67-1020.27  1013.95±7.99 | 839.19-886.49  865.89±19.79 |
|  | Ni | 0.0220-0.0222  0.0221±0.01 | 30.52-31.47  30.89±0.39 | 32.47-32.65  32.54±0.08 | 1387.27-1423.98  1402.40±15.66 | 1470.72-1475.91  1472.71±2.29 |
|  | Pb | 0.0233-0.0234  0.0234±0.01 | 63.15-63.55  63.39±0.17 | 59.05-59.44  59.19±0.18 | 2710.30-2715.81  2712.84±2.27 | 2523.50-2551.07  2532.98±12.80 |
|  | Fe | 0.0365-0.0366  0.0365±0.01 | 1012.33-1014.49  1013.43±0.88 | 1244.85-1256.66  1252.12±5.19 | 27718.31-27766.58  27739.98±20.01 | 34105.48-34429.04  34273.35±132.37 |
|  | Mn | 0.4743-0.4745  0.4744±0.01 | 2214.63-2217.59  2215.90±1.24 | 2654.95-2655.74  2655.48±0.37 | 4669.26-4673.53  4670.95±1.85 | 5595.26-5599.28  5597.55±1.69 |
|  | pH | 7.39-7.40  7.39±0.01 |  |  |  |  |
|  | electrolytic conductivity (µS∙cm^­-3^) | 1755.00-1756.00  1755.67±0.47 |  |  |  |  |
| no 5  pH 7.54 | Al | 0.2297-0.2298  0.2297±0.01 | 520.12-521.74  520.99±0.67 | 287.89-288.94  288.43±0.43 | 2263.36-2271.40  2267.82±3.34 | 1253.33-1257.35  1255.51±1.66 |
|  | Cu | 0.0193-0.0194  0.0193±0.02 | 71.82-71.84  71.83±0.01 | 44.46-44.48  44.47±0.01 | 3703.09-3721.76  3715.37±8.68 | 2291.75-2304.66  2300.01±5.86 |
|  | Cd | 0.0056-0.0057  0.0056±0.01 | 7.61-7.62  7.61±0.01 | 7.62-7.63  7.62±0.01 | 1336.84-1360.71  1352.16±10.86 | 1338.60-1360.71  1353.34±10.43 |
|  | Ni | 0.0217-0.0218  0.0217±0.01 | 31.70-31.72  31.71±0.01 | 32.14-32.15  32.15±0.01 | 1455.05-1461.29  1459.06±2.84 | 1474.77-1481.99  1478.99±2.99 |
|  | Pb | 0.0265-0.0266  0.0265±0.01 | 63.14-63.17  63.15±0.01 | 60.97-60.98  60.97±0.01 | 2374.06-2383.77  2380.16±4.34 | 2292.48-2300.75  2297.99±3.90 |
|  | Fe | 0.0260-0.0263  0.0261±0.01 | 1912.24-1912.26  1912.25±0.01 | 1566.47-1566.48  1566.47±0.01 | 72708.75-73548.08  73174.50±348.80 | 59561.60-60248.85  59942.94±285.59 |
|  | Mn | 0.0148-0.0149  0.0148±0.01 | 4649.61-4649.63  4649.62±0.01 | 4005.49-4005.51  4005.50±0.01 | 312055.03-314164.19  313460.69±993.95 | 268826.17-270641.89  270036.43±855.78 |
|  | pH | 7.61-7.62  7.61±0.01 |  |  |  |  |
|  | electrolytic conductivity (µS∙cm^­-3^) | 718.00-719.00  718.33±0.47 |  |  |  |  |
| no 6  pH 8.32 | Al | 0.2412-0.2413  0.2412±0.01 | 460.71-461.09  460.96±0.18 | 235.29-236.44  235.81±0.48 | 1910.07-1911.61  1910.85±0.63 | 975.50-979.86  977.50±1.80 |
|  | Cu | 0.0191-0.0192  0.0191±0.01 | 55.94-55.96  55.95±0.01 | 36.90-36.92  36.91±0.01 | 2913.54-2929.84  2924.23±7.56 | 1922.39-1932.98  1929.11±4.76 |
|  | Cd | 0.0061-0.0062  0.0061±0.01 | 7.33-7.35  7.34±0.01 | 8.44-8.46  8.45±0.01 | 1182.26-1204.92  1196.82±10.32 | 1362.90-1386.89  1377.79±10.62 |
|  | Ni | 0.0222-0.0223  0.0223±0.01 | 29.12-29.14  29.13±0.01 | 33.03-33.05  33.04±0.01 | 1306.28-1311.71  1308.24±2.46 | 1481.61-1487.84  1483.84±2.83 |
|  | Pb | 0.0183-0.0184  0.0183±0.01 | 55.34-55.36  55.35±0.01 | 67.77-67.79  67.78±0.01 | 3008.69-3024.59  3019.11±7.37 | 3683.15-3704.37  3697.11±9.88 |
|  | Fe | 0.0270-0.0272  0.0271±0.01 | 1754.85-1754.86  1754.85±0.01 | 1847.93-1847.94  1847.94±0.01 | 64516.54-64994.44  64755.32±195.10 | 67938.97-68442.22  68190.16±205.45 |
|  | Mn | 0.0142-0.0143  0.0143±0.01 | 569.83-569.85  569.84±0.01 | 498.55-498.58  498.56±0.01 | 39848.25-40130.28  39942.49±132.79 | 34864.34-35109.15  34946.41±115.08 |
|  | pH | 7.92-7.93  7.92±0.01 |  |  |  |  |
|  | electrolytic conductivity (µS∙cm^­-3^) | 863.00-864.00  863.33±0.47 |  |  |  |  |
| no 7  pH 9.11 | Al | 0.2612-0.2613  0.2613±0.01 | 750.08-751.55  750.88±0.61 | 238.11-239.55  238.87±0.59 | 2871.67-2876.20  2874.01±1.85 | 911.25-917.11  914.26±2.40 |
|  | Cu | 0.0178-0.0179  0.0178±0.01 | 83.15-83.18  83.17±0.01 | 66.61-66.63  66.62±0.01 | 4645.25-4673.03  4663.59±12.96 | 3722.35-3742.69  3735.73±9.46 |
|  | Cd | 0.0036-0.0037  0.0037±0.01 | 9.25-9.28  9.27±0.01 | 7.32-7.33  7.33±0.01 | 2500.00-2575.00  2527.70±33.61 | 1981.08-2033.33  1998.50±24.63 |
|  | Ni | 0.0182-0.0183  0.0183±0.01 | 43.15-43.16  43.15±0.01 | 34.84-34.88  34.86±0.02 | 2357.92-2370.88  2362.42±5.98 | 1903.83-1915.93  1908.59±5.27 |
|  | Pb | 0.0183-0.0184  0.0183±0.01 | 78.43-78.45  78.44±0.01 | 59.07-59.08  59.08±0.01 | 4262.50-4286.88  4278.57±11.37 | 3210.33-3228.42  3222.39±8.53 |
|  | Fe | 0.0242-0.0244  0.0243±0.01 | 2254.56-2254.59  2254.58±0.01 | 2547.41-2547.51  2547.45±0.04 | 92400.82-93163.64  92781.98±311.42 | 104402.46-105264.88  104834.38±352.08 |
|  | Mn | 0.0148-0.0150  0.0149±0.01 | 469.31-469.33  469.32±0.01 | 398.41-398.42  398.41±0.01 | 31287.33-31710.81  31498.93±172.88 | 26561.33-26919.59  26739.95±146.26 |
|  | pH | 7.43-7.44  7.43±0.01 |  |  |  |  |
|  | electrolytic conductivity (µS∙cm^­-3^) | 972.00-973.00  972.33±0.47 |  |  |  |  |

Table 6 Metal pollution index (MPI) in water and aquatic plants after the end of the experiment at week 7

| aquarium no / pH | Water | Branched bur-reed | River water-crowfoot |
| --- | --- | --- | --- |
| no1  pH 2.93 | 0.0720 | No plants | |
| no 2  pH 4.01 | 0.0404 | 99.62 | 123.41 |
| no 3  pH 5.39 | 0.0324 | 98.22 | 98.58 |
| no 4  pH 6.45 | 0.0525 | 140.44 | 133.74 |
| no 5  pH 7.54 | 0.0305 | 175.24 | 142.66 |
| no 6  pH 8.32 | 0.0292 | 117.28 | 106.14 |
| no 7  pH 9.11 | 0.0259 | 154.20 | 113.57 |

|  |  |
| --- | --- |
|  |  |
|  |  |
|  | Statistically significant differences are marked in the graph with letters a, b and in the table.  3 – pH 2.93; 4- pH 4.01; 5 – pH 5.39; 6 – pH 6.45; 7 – pH 7.54; 8 – pH 8.32; 9 – pH 9.11 |

Figure 3. Metal content of *S. erectum* after the experiment under varying water pH conditions

|  |  |
| --- | --- |
|  |  |
|  |  |
|  | Statistically significant differences are marked in the graph with letters a, b and in the table.  3 – pH 2.93; 4- pH 4.01; 5 – pH 5.39; 6 – pH 6.45; 7 – pH 7.54; 8 – pH 8.32; 9 – pH 9.11 |

Figure 4. Metal content of *R. fluitans* after the experiment under varying water pH conditions
